# Soil Carbon–Nitrogen Pools and Soil Health Across Contrasting Land Uses and a No‐Till Conservation Chronosequence in a Tropical Guinea Savanna Agroecosystem

**DOI:** 10.1002/pei3.70190

**Published:** 2026-07-17

**Authors:** Samuel Ayodele Mesele, Abosede Busayo Babatunde, Joseph Uponi

**Affiliations:** ^1^ International Institute of Tropical Agriculture (IITA) Ibadan Nigeria

**Keywords:** carbon storage, conservation agriculture, nutrient retention, organic matter fractions, tropical soils

## Abstract

Soil carbon–nitrogen pools are central to soil health, yet their responses to conservation‐oriented management remain insufficiently quantified in tropical agroecosystems. This study evaluated soil carbon–nitrogen pools, stocks and soil health indicators across conventionally managed arable land (CMA), no‐till‐based conservation systems with 2, 4, 7 and 10 years of establishment (NT2, NT4, NT7 and NT10), and natural forest land (NFL) in a tropical Guinea Savanna Ferric Luvisol in southwestern Nigeria. Soil samples were collected at 0–5 and 5–10 cm depths and analyzed for carbon and nitrogen fractions, stocks, physical properties, water‐regulation indicators and chemical fertility attributes. Total organic carbon ranged from 4.7 to 11.2 g kg^−1^, while total nitrogen ranged from 0.3 to 3.7 g kg^−1^ across land‐use systems and depths. Within the NT2–NT10 chronosequence, total nitrogen increased by 0.24 and 0.38 g kg^−1^ year^−1^ in the 0–5 and 5–10 cm layers, respectively, while nitrogen stock increased by 0.09 and 0.17 Mg ha^−1^ yr^−1^. Surface carbon stock also increased with no‐till duration by 0.21 Mg ha^−1^ year^−1^. Longer no‐till duration was associated with higher aggregate stability, increasing by 0.68 and 0.83 percentage units year^−1^ in the 0–5 and 5–10 cm layers, respectively. Soil pH increased by 0.15 and 0.13 units year^−1^, while cation exchange capacity increased by approximately 0.46 cmolc kg^−1^ year^−1^ in both depths. In contrast, leaching potential declined by 0.98 and 1.29 percentage units year^−1^ in the 0–5 and 5–10 cm layers, respectively. The integrated soil health score increased from 19.6 under NT2 to 82.6 under NT10 in the surface layer, and NT10 also had the highest score in the 5–10 cm layer. These findings indicate that sustained no‐till‐based conservation management can improve soil carbon–nitrogen status, nutrient retention, water regulation and overall soil health. However, the observed responses reflect the combined effects of reduced disturbance, organic inputs, mulching, perennial crop cover and irrigation rather than no‐till alone.

## 1 Introduction

Soil health remains central to sustainable agriculture and effective ecosystem management, shaping productivity, resilience, and environmental quality (Doran and Zeiss 2000; Bai et al. 2018; Topa et al. 2025). Among the many indicators used to evaluate soil health, soil organic carbon and nitrogen pools stand out for their direct roles in regulating soil structure, nutrient availability, and biological activity (Lal 2004; Topa et al. 2025; Kerketta et al. 2026). These pools are highly responsive to land use and management history, including tillage intensity, vegetation cover, and organic matter inputs, making them sensitive indicators of soil degradation or recovery (Aziz et al. 2013; Kan et al. 2022; Ansari et al. 2026).

Across West Africa, widespread conversion of natural ecosystems into agricultural land has resulted in substantial declines in soil carbon and nitrogen reserves (Mesele et al. 2025). Such losses are closely linked to imbalances in organic matter inputs and outputs, often driven by changes in vegetation composition and land management practices (Dawson and Smith 2007; Beillouin et al. 2022; Yang et al. 2025). Tillage‐based systems accelerate organic matter mineralization and disrupt soil aggregates, thereby weakening soil structure and function. These concerns are especially relevant for inherently fragile soils such as Oxic Paleustalfs, where the risk of soil organic matter depletion is high. In this context, there is growing interest in identifying management practices that can reverse degradation trends and restore soil function. No‐till (NT) agriculture has emerged as a promising alternative, attracting considerable attention for its potential to improve soil health through reduced disturbance and enhanced residue retention (Mangalassery et al. 2015; Jayaraman and Dalal 2022; Mondal and Chakraborty 2022; Srivastava 2025). This approach promotes the accumulation of organic matter, supports soil biodiversity, and improves soil structure. Evidence suggests that NT systems can positively influence soil carbon and nitrogen dynamics, as well as other key properties linked to soil fertility and ecosystem functioning (Turmel et al. 2015; Usharani et al. 2019; Srivastava 2025).

Different land use systems, including conventional tillage, no‐till agriculture, and natural forest vegetation, create distinct soil environments, each with unique implications for nutrient cycling and soil quality (Rotich et al. 2025). The duration of no‐till adoption adds another important dimension, as the benefits of reduced disturbance often develop gradually over time (Thomas et al. 2007; Aziz et al. 2013; Song et al. 2026). Despite growing recognition of these temporal effects, evidence remains limited for mesic savanna and forest ecologies, where climatic and soil conditions may influence the rate and extent of soil recovery. A clearer understanding of how land use and management duration interact to shape soil carbon and nitrogen pools is therefore essential for guiding sustainable land management. Particular attention is required for the different fractions of soil carbon, including particulate and non‐particulate forms, as these provide valuable information on both short‐term dynamics and long‐term stabilization processes. Such knowledge is critical for evaluating the sustainability of agricultural systems, especially in soils that are highly susceptible to organic matter loss (Mesele et al. 2016).

In this context, this study evaluates the effects of contrasting land use systems (conventionally managed land, no‐till systems of varying years, and natural forest land) on soil carbon and nitrogen pools and a suite of soil health indicators in a tropical guinea savanna. Specifically, the study assesses how the duration of no‐till management influences the recovery of soil properties and overall soil function, with natural forest conditions serving as a benchmark. A comprehensive evaluation of indicators, including bulk density, particle size distribution, soil moisture characteristics, pH, electrical conductivity, aggregate stability, leaching potential, and rooting depth, provides a holistic view of soil conditions under contrasting management systems. The findings are intended to inform land management strategies that strengthen soil resilience, enhance nutrient cycling, and support sustainable agricultural production. Beyond local relevance in Ibadan, Nigeria, the study offers insights applicable to other tropical regions with similar pedoclimatic conditions, contributing to broader efforts aimed at restoring soil‐based ecosystem functions and improving environmental sustainability.

## 2 Materials and Methods

### 2.1 Study Location

The research was conducted within the 1000‐ha land of the IITA Ibadan campus in Nigeria (Figure S1). Ibadan, located in southwestern Nigeria, has a tropical wet and dry climate. The rainy season lasts from March to October, peaking between June and September, with average annual rainfall between 1200 and 1500 mm. High humidity during this period supports lush vegetation, while the dry season from November to February features low humidity and little rainfall, with January known for the harmattan—a phase of dry, dusty winds from the Sahara. Average daily temperatures range from 20°C to 30°C (68°F to 86°F), with March and April being the hottest months. These climatic conditions make Ibadan a vital agricultural hub in Nigeria, enabling the successful cultivation of diverse crops.

### 2.2 Pedological Characteristics of the Study Area

The soils are derived from the Pre‐Cambrian Basement Complex rock, with banded gneiss being a common type. Specifically, these soils consist of gray quartz‐biotite plagioclase‐hornblende paragneiss. They are situated on upper convex slopes, well‐drained, and are classified as the Ibadan series. The epipedon is sandy, with a low percentage of silt and significant amounts of quartzite and quartz gravel. These soils have low water‐holding capacity and limited structural stability against raindrop impact. Therefore, practices that conserve moisture and protect the soil surface are often highly desirable for this type of soil. Quartz and kaolinite are the predominant soil minerals; thus, organic matter accounts for a large portion of the topsoil's cation exchange capacity. Any loss of soil organic matter due to inappropriate soil management could lead to severe soil degradation, in part due to increased nutrient leaching. The soils are classified as Oxic Paleustalf or Ferric Luvisol, in accordance with the USDA and FAO‐WRB classification systems, respectively. A full description of the characteristics of the soils of IITA Ibadan is presented in Huising and Mesele (2021).

### 2.3 Experimental Design and Land Use History

**FIGURE 1:**
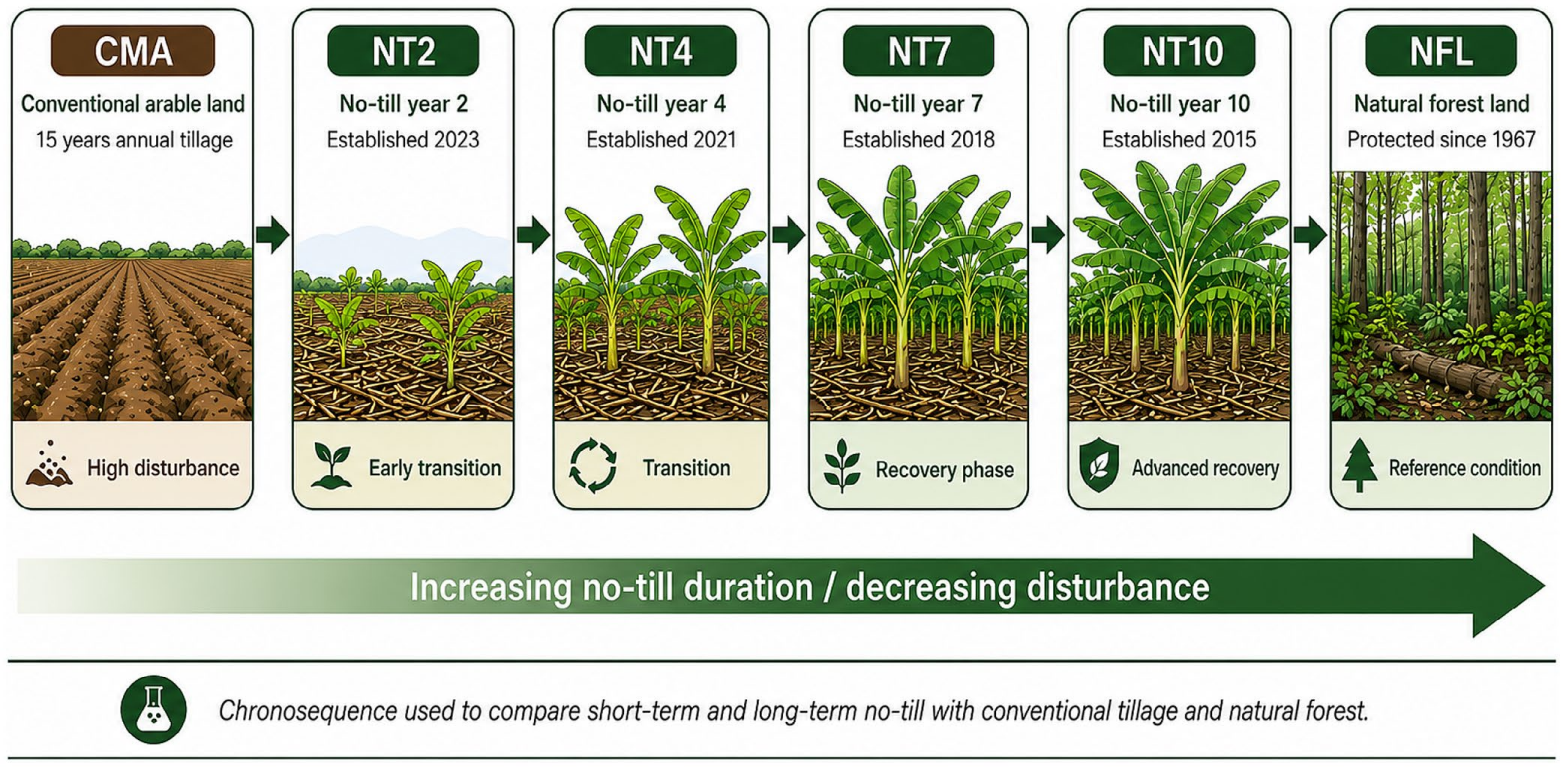
Land‐use and management‐history structure used to assess soil carbon–nitrogen pools and soil health indicators. The study compared conventionally managed arable land (CMA), four no‐till‐based conservation systems established for 2, 4, 7, and 10 years (NT2, NT4, NT7, and NT10), and natural forest land (NFL) as a reference condition. The horizontal arrow represents increasing no‐till duration and decreasing soil disturbance across the managed systems. The no‐till systems were interpreted as a chronosequence of integrated conservation management rather than as repeated temporal measurements of the same field.

The experiment was conducted at Ibadan, Nigeria (7.5032° N, 3.9034° E). It was originally designed as a banana varietal trial in 2015 under the Excellence in Breeding Initiative of the Consultative Group on International Agricultural Research (CGIAR). Plots (now referred to as NT) each measuring 12 × 8 rows were laid out in a randomized complete block design with three replications on a gently sloping terrain with similar soil characteristics. Each block, therefore, represented a true field‐level replicate within the original experimental design. Subsequent field trials were established in 2018, 2021, and 2023, a few meters away from the 2015 field. These fields, now referred to as NT2, NT4, NT7, and NT10, represent a chronosequence (space‐for‐time substitution) approach, where each no‐till system corresponds to an independent field site established in different years under comparable soil and environmental conditions. The study compared conventionally managed arable land, four no‐till‐based conservation systems with differing establishment durations, and natural forest land as a reference condition (Figure 1). In this context, each land‐use treatment represents a distinct field site rather than repeated measurements of a single plot over time. Similar agronomic management, including mulching, weeding, irrigation, and organic fertilization, was carried out on all fields, irrespective of the year of planting. Adjacent to these banana trial fields are conventionally managed arable lands, with the same soil type, but different management practices. About two kilometers away is the natural forest land (NFL), which is more of a secondary forest that has been protected since 1967. The detailed, specific land‐use history and land management for the various land types were reported by Mesele et al. (2026). In January 2025, a soil inventory and land use assessment were conducted to evaluate soil organic carbon (including its different fractions) and other soil health indicators, vis‐à‐vis the various management practices over varying periods. Given the close spatial proximity of the sites, potential spatial variability may influence the observed differences and the interpretation of temporal trends across the chronosequence.

### 2.4 Soil Sampling Campaign

Soil samples were collected using a randomized sampling approach in each land use type. Four composite samples were collected in two groups for each land use type. The Y‐frame sampling approach was adopted for the composite sampling (Huising and Mesele 2021, 2022). A cylindrical core of 100 cm^3^ was used to collect undisturbed soil samples from each land use type at two depths: 0–5 cm (topsoil) and 5–10 cm (subsoil). Due to the shallow nature of the soils, sampling could not be conducted beyond 30 cm because of soil depth restrictions caused by iron pans and stone concretions. Since no‐till effects are typically concentrated in the surface layer, this depth limitation affects the interpretation of the results, implying that the findings are more representative of the topsoil than of subsoil conditions in most tropical soils. Collected soil samples were neatly packed in well‐labeled, QR‐coded bags and sent to the analytical service laboratory at IITA for sample preparation. Quality control procedures were implemented throughout the laboratory analyses. Instruments were calibrated using appropriate standards before analysis, and blank samples were analyzed to monitor potential contamination during the laboratory procedures. Selected samples were analyzed in duplicate to assess precision, and measurements were repeated when discrepancies were found. All analyses were conducted following standard laboratory protocols.

### 2.5 Soil Organic Carbon Fractionation

Particulate soil organic carbon (POC) is separated into fractions with diameters ranging from 53 to 250 μm and quantified by wet sieving. Following the protocol established by Cambardella and Elliott (1994), particulate organic matter (POM) was isolated from the soil mineral matrix. Specifically, 50 g of fresh soil samples were treated with 50 mL of 5 g/L sodium hexametaphosphate and agitated for 16 h on a reciprocal shaker, followed by wet sieving to minimize alterations to the soil's structural integrity that often result from air or oven drying. The dispersed soil was subsequently passed through a series of sieves: 2 mm, 250 μm, and 53 μm. The retained fractions, coarse particulate organic carbon (POCc) between 2 mm and 250 μm, fine particulate organic carbon (POCf) between 250 μm and 53 μm, and non‐particulate organic carbon (< 53 μm), were collected and subjected to oven drying at 65°C for 24 h. The mass of each dried sample was recorded. To assess the organic carbon content of these fractions, the Walkley‐Black wet oxidation method, as delineated by Walkley and Black (1934), was employed. Concurrently, the nitrogen (N) content of each fraction was determined using a modified Kjeldahl approach, as specified by Bremner and Mulvaney (1982).

### 2.6 Assessment of Soil Carbon and Nitrogen Stocks

Soil bulk density was measured using the gravimetric method (Blake 1965) at each sampling depth to convert SOC concentration into carbon stocks (Mg ha^−1^). The SOC stocks were calculated using the formula:

$$\begin{array}{c} \mathrm{SOC}\;\text{stock}\;\left(\mathrm{Mg}\;{\mathrm{ha}}^{-1}\right)=\mathrm{SOC}\;\text{concentration}\;\left(g\;{\mathrm{kg}}^{-1}\right)\\ \;\times \text{bulk density}\;\left(g\;{\mathrm{cm}}^{-3}\right)\times \text{depth}\;\left(\mathrm{cm}\right)\times 0.1 \end{array}$$

SOC concentration in g kg^−1^, bulk density in g cm^−3^ and depth in cm. SOC stock was expressed in Mg ha^−1^. Nitrogen stocks were calculated similarly to SOC using the nitrogen values.

### 2.7 Evaluation of Soil Health Indicators

Soil health indicators were evaluated through a series of physical and chemical analyses. The particle size distribution was assessed via the hydrometer method (Bouyoucos 1962), while bulk density was quantified using the core method, defined as the mass of oven‐dried soil per unit volume of the sampled soil (Blake 1965). Fine‐earth bulk density was determined using a modified approach in which coarse fragments were removed to obtain true bulk density values (Blake 1965). Soil pH and electrical conductivity (EC) were measured in a 1:2 soil–H_2_O suspension using an electronic pH meter, following the protocols established by Smith and Doran (1997). Available phosphorus was quantified using the Bray‐1 extraction solution, as outlined by Bray and Kurtz (1945). Hydraulic conductivity was assessed using the model proposed by Abdelbaki (2021).

Soil aggregate stability was determined following the methodology of Asadi and Bagheri (2010). The available water‐holding capacity and field capacity were determined in accordance with the guidelines of Mtama et al. (2018). Rooting depth metrics were sourced from work by Bayabil et al. (2019), whereas the permanent wilting point was evaluated using Mtama et al. (2018) methods. Leaching potential was derived from the model established by Shea et al. (1992). Finally, cation exchange capacity served as a proxy for nutrient retention capacity, as measured using the framework proposed by Mtama et al. (2018).

### 2.8 Data Analyses

All statistical analyses were conducted in R (Version 4.6.0). The dataset was first checked for completeness, unit consistency, coding errors, and possible outliers. Land‐use systems were coded as conventionally managed arable land (CMA), 2‐year no‐till system (NT2), 4‐year no‐till system (NT4), 7‐year no‐till system (NT7), 10‐year no‐till system (NT10), and natural forest land (NFL). Soil depth was coded as 0–5 cm and 5–10 cm. Results are presented as means with standard errors unless otherwise stated. The effects of land use, soil depth, and their interaction on soil carbon–nitrogen pools, stocks, C:N ratio, and soil health indicators were tested using block‐adjusted linear models. Land use, soil depth, and the land use × depth interaction were treated as fixed effects, while sampling replicate was included as a blocking term. The general model structure was:

Response variable~landuse×soil depth+replicate.

where land use × depth interactions were relevant, land‐use comparisons were interpreted within each soil depth. Mean separation among land‐use systems within each depth was performed using estimated marginal means followed by Tukey‐adjusted pairwise comparisons at *p* < 0.05. For the within‐depth comparisons used to assign significance letters, the model structure was:

Response variable~landuse+replicate.

Model assumptions were assessed using residual‐versus‐fitted plots, quantile–quantile plots, the Shapiro–Wilk test for residual normality and Levene's test for homogeneity of variance. Variables were transformed where necessary to improve model assumptions. Exact *p*‐values are reported where appropriate. To evaluate directional changes within the no‐till systems, chronosequence trend analysis was conducted using NT2, NT4, NT7, and NT10 only. No‐till duration was treated as a continuous predictor, while soil depth and replicate were retained in the model. The model structure was:

Response variable~no−till duration×soil depth+replicate.

Chronosequence slopes were estimated as the change in each soil property per additional year of no‐till duration. Slope estimates and 95% confidence intervals are reported in Table S1. Because the no‐till systems were separate fields established in different years, these slopes were interpreted as associations along a space‐for‐time management gradient rather than repeated temporal changes in the same field.

An integrated soil health score was calculated using selected carbon–nitrogen and soil functional indicators: total organic carbon, total nitrogen, non‐particulate organic carbon, non‐particulate nitrogen, carbon stock, nitrogen stock, cation exchange capacity, aggregate stability, available water‐holding capacity, and field capacity. Each indicator was rescaled from 0 to 100 using min–max normalization within each soil depth, with higher scores representing more favorable relative soil conditions. The composite score for each land‐use system and depth was calculated as the mean of the normalized indicator scores. Texture variables, bulk density, leaching potential, and rooting depth were excluded from the integrated score to avoid combining inherent site characteristics or indicators with ambiguous functional direction.

Pearson correlation analysis was used to assess pairwise associations among soil carbon–nitrogen pools, stocks, and soil health indicators. Principal component analysis (PCA) was used as an exploratory multivariate approach to summarize the main gradients of variation among soil properties and land‐use systems. Variables included in the PCA were standardized to zero mean and unit variance before analysis to account for differences in measurement units. Particle‐size distribution was treated as a background site‐characterization variable.

## 3 Results

### 3.1 Soil Carbon–Nitrogen Pools and Stocks

Soil carbon and nitrogen pools varied among land‐use systems and soil depths (Table 1). Across both soil layers, total organic carbon (TOC) ranged from 4.7 to 11.2 g kg^−1^, while total nitrogen (TN) ranged from 0.3 to 3.7 g kg^−1^. In the 0–5 cm layer, TOC was relatively similar across land‐use systems, ranging from 9.2 g kg^−1^ under CMA to 11.2 g kg^−1^ under NT4. In the 5–10 cm layer, TOC showed stronger separation among land uses, with the lowest value under CMA and the highest value under NFL. Within the no‐till systems, chronosequence trend analysis showed significant positive duration effects for TN, POCt, PONt, carbon stock and nitrogen stock in the 0–5 cm layer, and for TOC, TN and nitrogen stock in the 5–10 cm layer (Table S1). TOC increased with no‐till duration in the 5–10 cm layer, with a significant positive duration trend of 0.30 g kg^−1^ year^−1^. Total nitrogen showed clearer differentiation among land uses than TOC. In the 0–5 cm layer, TN was lowest under CMA and highest under NT10, increasing from 1.15 g kg^−1^ under NT2 to 3.73 g kg^−1^ under NT10. In the 5–10 cm layer, TN was also highest under NT10, followed by NT7 and NFL. The chronosequence analysis showed a significant positive effect of no‐till duration on TN in both the 0–5 cm and 5–10 cm layers, with slopes of 0.24 and 0.38 g kg^−1^ year^−1^, respectively.

**TABLE 1:** Soil carbon–nitrogen pools and stocks under contrasting land‐use systems and no‐till duration.

Soil layer	Land use	TOC	TN	POCt	PONt	nPOC	nPON	C stock	N stock	C:N
0–5 cm	CMA	9.2ᵃ	0.47ᶠ	15.0ᵃ	12.9ᵇ	7.4ᵃᵇ	13.4ᵇ	4.81ᵃᵇ	0.25ᵉ	19.5ᵃ
0–5 cm	NT2	9.3ᵃ	1.15ᵉ	3.8ᶜ	1.2ᶠ	7.4ᵃᵇ	1.6ᶠ	4.53ᵇ	0.81ᵈ	8.1ᶜ
0–5 cm	NT4	11.2ᵃ	3.44ᵇ	0.7ᵈ	6.9ᵈ	2.5ᵇ	7.6ᶜ	5.27ᵃᵇ	1.62ᵇ	3.3ᵈ
0–5 cm	NT7	10.4ᵃ	2.73ᶜ	2.3ᶜᵈ	9.6ᶜ	10.3ᵃ	2.9ᵉ	6.04ᵃ	1.58ᵇ	3.8ᵈ
0–5 cm	NT10	10.8ᵃ	3.73ᵃ	10.5ᵇ	31.5ᵃ	7.0ᵃᵇ	4.7ᵈ	6.16ᵃ	1.70ᵃ	15.3ᵇ
0–5 cm	NFL	10.0ᵃ	2.18ᵈ	14.5ᵃ	4.7ᵉ	8.5ᵃᵇ	16.7ᵃ	4.55ᵇ	0.99ᶜ	4.6ᵈ
0–5 cm	*p*‐value	0.128	< 0.001	< 0.001	< 0.001	0.034	< 0.001	0.015	< 0.001	< 0.001
5–10 cm	CMA	4.7ᵈ	0.55ᵈ	2.5ᵃᵇ	2.4ᶜ	0.9ᵈ	2.9ᵉ	2.71ᶜ	0.32ᵈ	8.6ᵇ
5–10 cm	NT2	6.6ᶜᵈ	0.33ᶠ	0.5ᵇ	1.8ᵉ	1.8ᵈ	2.1ᶠ	3.05ᵇᶜ	0.20ᶠ	8.8ᵇ
5–10 cm	NT4	7.9ᵇᶜ	0.51ᵉ	2.8ᵃᵇ	1.0ᶠ	6.5ᵇᶜ	4.3ᵇ	4.04ᵃᵇ	0.26ᵉ	15.4ᵃ
5–10 cm	NT7	9.7ᵃᵇ	2.16ᵇ	2.2ᵃᵇ	2.5ᵇ	7.5ᵃᵇ	4.2ᶜ	4.18ᵃᵇ	1.27ᵇ	4.5ᶜ
5–10 cm	NT10	8.8ᵃᵇᶜ	3.16ᵃ	1.7ᵃᵇ	1.9ᵈ	4.9ᶜ	3.6ᵈ	3.72ᵃᵇᶜ	1.37ᵃ	13.5ᵃ
5–10 cm	NFL	10.5ᵃ	2.01ᶜ	7.3ᵃ	5.2ᵃ	8.6ᵃ	5.3ᵃ	4.62ᵃ	0.89ᶜ	5.2ᶜ
5–10 cm	*p*‐value	0.002	< 0.001	0.037	< 0.001	< 0.001	< 0.001	0.009	< 0.001	< 0.001

*Note:* Values are means. TOC and TN are expressed in g kg^−1^. POCt, PONt, nPOC and nPON are in grams (g). Multiply values of POCt, PONt and nPON by 0.1 to get the actual concentrations. Carbon stock and nitrogen stock are expressed in Mg ha^−1^. C:N is dimensionless. Within each soil layer and variable, means followed by the same superscript letter are not significantly different based on block‐adjusted Tukey comparisons at *p* < 0.05. The *p*‐value rows indicate the land‐use effect within each soil depth from block‐adjusted ANOVA.

Abbreviations: CMA, conventionally managed arable land; NFL, natural forest land; nPOC, non‐particulate organic carbon; nPON, non‐particulate nitrogen; NT10, 10‐year no‐till system; NT2, 2‐year no‐till system; NT4, 4‐year no‐till system; NT7, 7‐year no‐till system; POCt, total particulate organic carbon; PONt, total particulate nitrogen; TN, total nitrogen; TOC, total organic carbon.

Total particulate organic carbon (POCt) showed a pattern that contrasted with TOC. In the 0–5 cm layer, POCt was highest under CMA and NFL, followed by NT10, and lower under NT2, NT4, and NT7. In the 5–10 cm layer, POCt was highest under NFL and lowest under NT2. Within the no‐till systems, POCt increased significantly with no‐till duration in the 0–5 cm layer, with a slope of 0.088 g C year^−1^. Total particulate nitrogen (PONt) was highest under NT10 in the 0–5 cm layer and highest under NFL in the 5–10 cm layer. The increase in PONt with no‐till duration was significant in the 0–5 cm layer, with a slope of 0.352 g C year^−1^, but not in the 5–10 cm layer. Non‐particulate organic carbon (nPOC) and non‐particulate nitrogen (nPON) differed among land‐use systems, but their responses were less consistently aligned with no‐till duration. In the 0–5 cm layer, nPOC was highest under NT7 and lowest under NT4, while in the 5–10 cm layer, the highest values occurred under NFL and NT7. Non‐particulate nitrogen was highest under NFL in both soil layers. The chronosequence trend analysis did not show significant directional changes in nPOC or nPON with increasing no‐till duration. Detailed coarse‐ and fine‐particulate organic carbon and nitrogen fractions are provided in Table S2.

Carbon stocks ranged from 4.53 to 6.16 Mg ha^−1^ in the 0–5 cm layer and from 2.71 to 4.62 Mg ha^−1^ in the 5–10 cm layer. In the surface layer, the highest carbon stocks were observed under NT7 and NT10, whereas NT2 and NFL had lower values. In the 5–10 cm layer, NFL had the highest carbon stock, followed by NT7 and NT4. Carbon stock increased significantly with no‐till duration in the 0–5 cm layer, with a slope of 0.20 Mg ha^−1^ year^−1^. Nitrogen stocks ranged from 0.25 to 1.70 Mg ha^−1^ in the 0–5 cm layer and from 0.20 to 1.37 Mg ha^−1^ in the 5–10 cm layer. In both soil layers, NT10 had the highest nitrogen stock, while CMA had the lowest value in the surface layer and NT2 had the lowest value in the 5–10 cm layer. Nitrogen stock increased significantly with no‐till duration in both soil layers, with slopes of 0.09 Mg ha^−1^ year^−1^ in the 0–5 cm layer and 0.17 Mg ha^−1^ year^−1^ in the 5–10 cm layer. The C:N ratio ranged from 3.25 to 19.50 in the 0–5 cm layer and from 4.50 to 15.40 in the 5–10 cm layer, but no consistent chronosequence trend was detected.

### 3.2 Multivariate Relationships Among Soil Health Indicators

**FIGURE 2:**
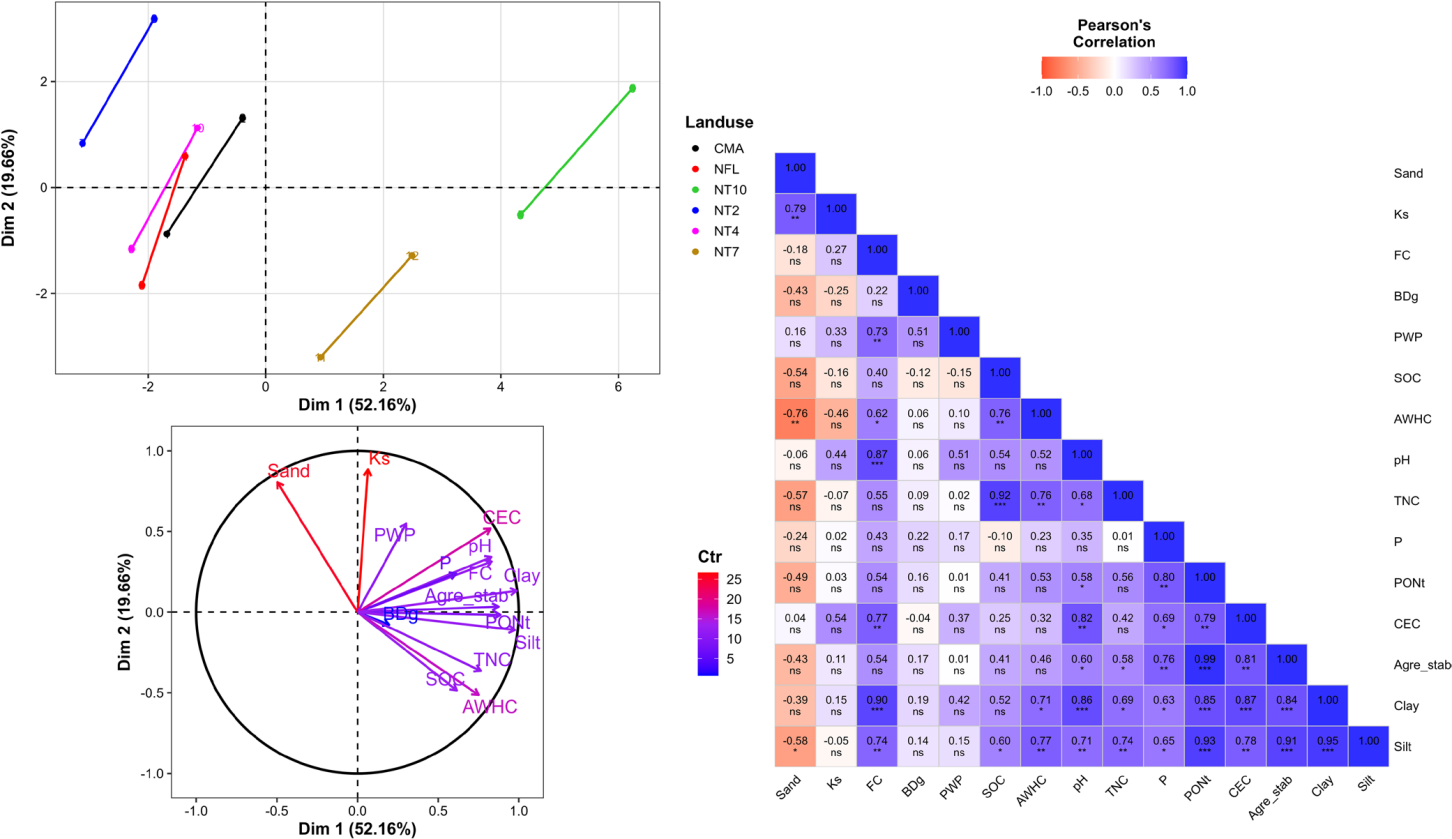
Principal component analysis and correlation matrix of the soil health indicators. Agre_stab, aggregate stability; AWHC, available water holding capacity; BDg, bulk density of the whole soil earth; FC, field capacity; Ks, saturated hydraulic conductivity; POCt, total particulate soil organic carbon; PONt, total particulate nitrogen; PWP, permanent wilting point; SOC, soil organic carbon stock; TNC, total nitrogen stock.

The first two PCA dimensions explained 71.82% of the total variation among soil health indicators, with Dimension 1 and Dimension 2 accounting for 52.16% and 19.66%, respectively (Figure 2). Dimension 1 mainly represented a soil fertility and structural gradient associated with CEC, pH, available phosphorus, aggregate stability, clay and silt, while sand and saturated hydraulic conductivity were positioned in the opposite direction. Dimension 2 reflected variation associated mainly with hydraulic and physical indicators, including saturated hydraulic conductivity, sand and permanent wilting point. The correlation matrix showed strong positive associations among CEC, available phosphorus, pH, aggregate stability, clay and silt. Saturated hydraulic conductivity was positively associated with sand and negatively associated with available water‐holding capacity.

### 3.3 Soil Physical and Water‐Regulation Indicators

**FIGURE 3:**
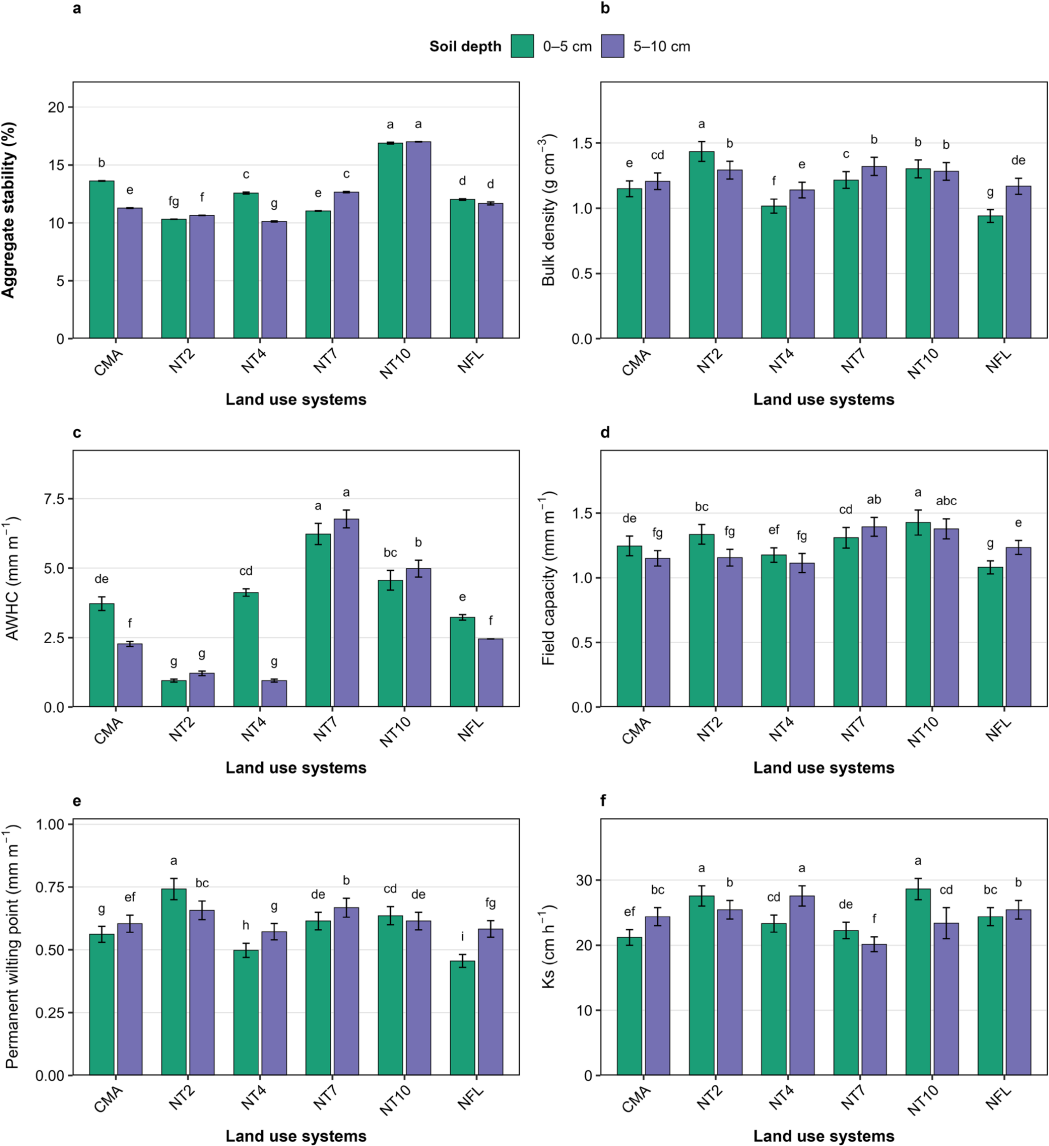
Soil physical and water‐regulation indicators across contrasting land‐use systems and soil depths. Panels show (a) aggregate stability, (b) bulk density, (c) available water‐holding capacity, (d) field capacity, (e) permanent wilting point, and (f) saturated hydraulic conductivity. Bars represent means and error bars indicate standard errors. Different letters indicate significant differences among land‐use systems within the same soil depth based on block‐adjusted Tukey comparisons at *p* < 0.05. CMA, conventionally managed arable land; NFL, natural forest land; NT2, 2‐year no‐till system; NT4, 4‐year no‐till system; NT7, 7‐year no‐till system; NT10, 10‐year no‐till system.

Particle size distribution is presented as a supplementary site‐characterization result (Figure S2). Sand, silt, and clay contents differed among land‐use systems and soil depths, showing background textural variability among the sampled fields. These texture data are presented separately from the main soil health response indicators. Soil physical and water‐regulation indicators showed clear differences among land‐use systems and between soil depths (Figure 3). Aggregate stability ranged from approximately 10 to 17% across the land‐use systems and was highest under NT10 in both the 0–5 cm and 5–10 cm layers (Figure 3a). The remaining land‐use systems, including CMA, NT2, NT4, NT7, and NFL, had lower aggregate stability values than NT10. Aggregate stability increased significantly with no‐till duration in both soil layers, with slopes of 0.68% year^−1^ in the 0–5 cm layer and 0.83% year^−1^ in the 5–10 cm layer. Chronosequence trend slopes for the no‐till systems are provided in Table S1. Bulk density showed a different pattern from aggregate stability (Figure 3b). In the 0–5 cm layer, the highest bulk density was observed under NT2, whereas NFL had the lowest. CMA, NT4, NT7, and NT10 had intermediate values. In the 5–10 cm layer, differences among land‐use systems were less pronounced, although NT2 and NT7 maintained relatively higher values than NFL.

Water‐retention indicators more clearly separated the longer‐duration no‐till systems from the other land‐use systems. Available water‐holding capacity was highest under NT7 and NT10 in both soil layers and lower under CMA, NT2, and NFL (Figure 3c). Field capacity followed a broadly similar pattern, with higher values under NT7 and NT10 than under the other land‐use systems (Figure 3d). Permanent wilting point differed among land‐use systems and soil depths, but the pattern was less consistent than those observed for available water‐holding capacity and field capacity (Figure 3e). Saturated hydraulic conductivity ranged from approximately 18 to 28 cm h^−1^, with no consistent directional pattern across the no‐till duration sequence (Figure 3f). Rooting depth and leaching potential are presented as supplementary physical indicators (Figure S3). Rooting depth ranged from approximately 14 to 20 cm, with lower values under NT2 and higher values under CMA and NT10. Leaching potential ranged from approximately 40 to 72%, with lower values under NT7 and NT10 and higher values under CMA and NFL. Leaching potential declined significantly with increasing no‐till duration in both soil layers, with slopes of −0.98% year^−1^ in the 0–5 cm layer and −1.29% year^−1^ in the 5–10 cm layer.

### 3.4 Soil Chemical Fertility Indicators

**FIGURE 4:**
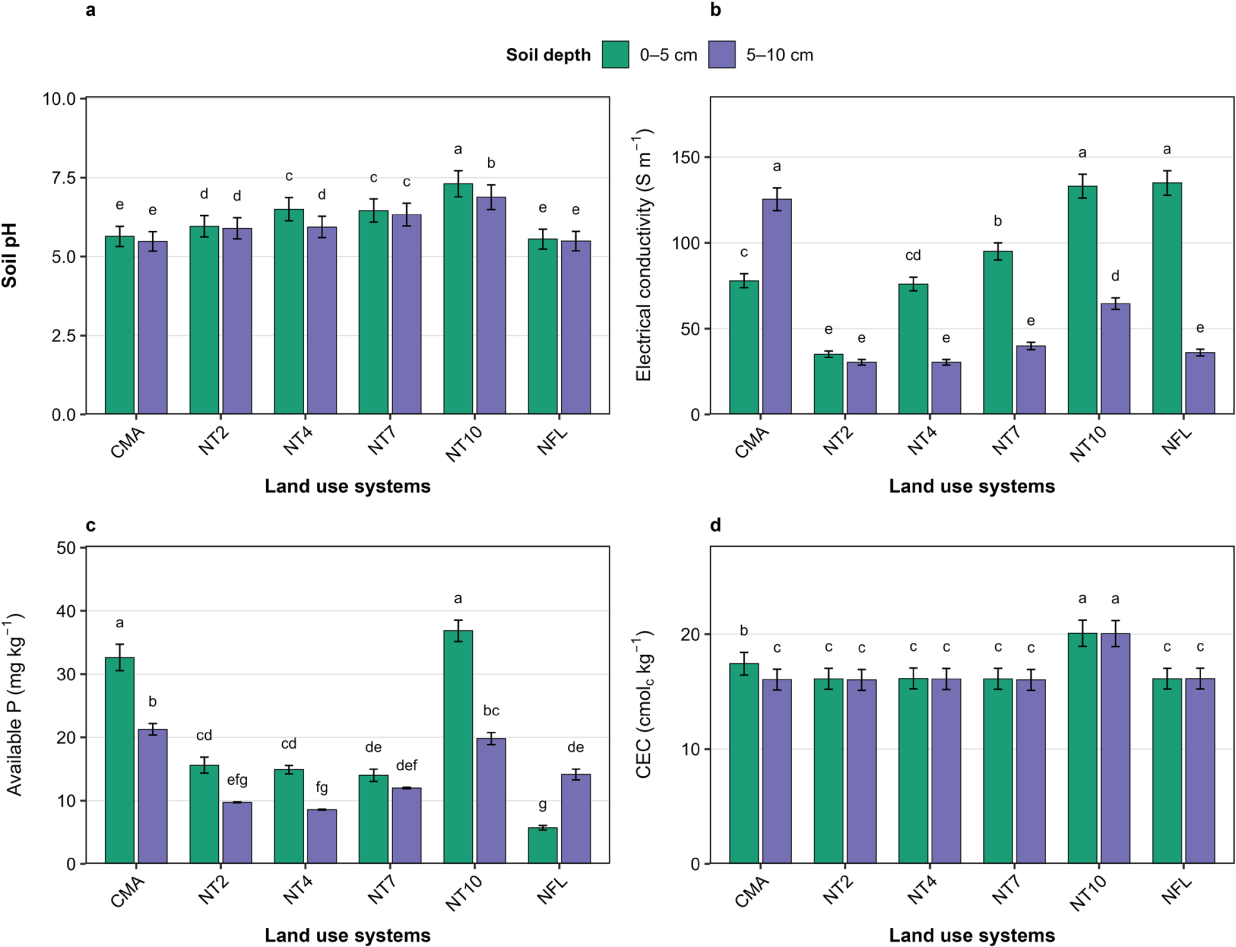
Soil chemical fertility indicators across contrasting land‐use systems and soil depths. Panels show (a) soil pH, (b) electrical conductivity, (c) available phosphorus, and (d) cation exchange capacity. Bars represent means and error bars indicate standard errors. Different letters indicate significant differences among land‐use systems within the same soil depth based on block‐adjusted Tukey comparisons at *p* < 0.05. CMA, conventionally managed arable land; CEC, cation exchange capacity; EC, electrical conductivity; NFL, natural forest land; NT2, 2‐year no‐till system; NT4, 4‐year no‐till system; NT7, 7‐year no‐till system; NT10, 10‐year no‐till system.

Soil chemical fertility indicators also differed among land‐use systems and soil depths (Figure 4). Soil pH ranged from moderately acidic to near neutral, with higher values generally recorded under the no‐till systems than under CMA and NFL (Figure 4a). Among the no‐till systems, NT10 had the highest pH values in both soil layers. Within the no‐till systems, pH increased significantly with duration in both soil layers, with slopes of 0.15 and 0.13 pH units year^−1^ in the 0–5 cm and 5–10 cm layers, respectively. Electrical conductivity showed depth‐dependent variation across land‐use systems (Figure 4b). In the 0–5 cm layer, EC was lowest under NT2 and higher under NT7, NT10, and NFL. In the 5–10 cm layer, the highest EC value was observed under CMA, whereas most no‐till systems had lower values. Available phosphorus showed strong differentiation among land‐use systems, especially in the surface layer (Figure 4c). In the 0–5 cm layer, NT10 had the highest available phosphorus concentration, followed by CMA, whereas NFL had the lowest value. Available phosphorus concentrations were generally lower in the 5–10 cm layer than in the 0–5 cm layer across most land‐use systems. No‐till duration had a significant positive effect on available P in the 0–5 cm layer, with a slope of 2.44 mg kg^−1^ year^−1^, while the positive trend in the 5–10 cm layer was weaker. Cation exchange capacity ranged from approximately 15 to 20 cmolc kg^−1^ and showed the clearest separation under NT10 (Figure 4d). The highest CEC values were recorded under NT10, particularly in the 5–10 cm layer, while the remaining land‐use systems had relatively similar values.

### 3.5 Integrated Soil Health Score

**FIGURE 5:**
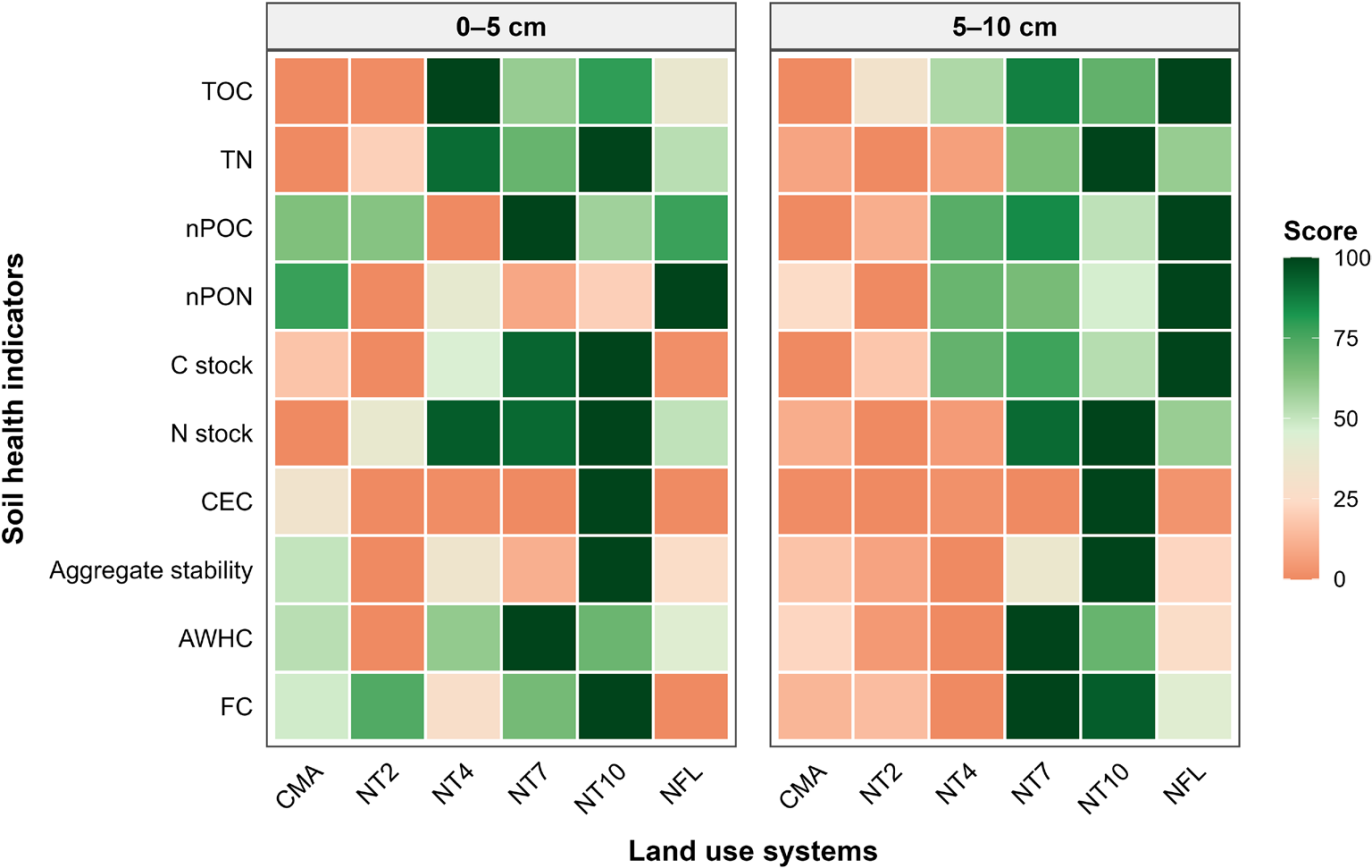
Integrated soil health score across contrasting land‐use systems and soil depths. Scores were calculated from selected soil carbon–nitrogen and soil health indicators using min–max normalization within each soil depth. Higher scores indicate relatively more favorable soil conditions for each indicator. The indicators included total organic carbon (TOC), total nitrogen (TN), non‐particulate organic carbon (nPOC), non‐particulate nitrogen (nPON), carbon stock, nitrogen stock, cation exchange capacity (CEC), aggregate stability, available water‐holding capacity (AWHC), and field capacity (FC). Red colors indicate lower relative scores, while green colors indicate higher relative scores. CMA, conventionally managed arable land; NFL, natural forest land; NT2, 2‐year no‐till system; NT4, 4‐year no‐till system; NT7, 7‐year no‐till system; NT10, 10‐year no‐till system.

The integrated soil health score provided a summary of the relative performance of selected carbon–nitrogen and soil functional indicators across land‐use systems and soil depths (Figure 5). In the 0–5 cm layer, NT10 showed consistently high scores for several indicators, including TN, nPOC, nPON, C stock, N stock, CEC, and field capacity. NT7 also showed high scores for water‐regulation indicators, particularly available water‐holding capacity. In contrast, CMA and NT2 had lower scores for several indicators. In the 5–10 cm layer, the score pattern was less uniform than in the surface layer. Higher scores were observed under NT7, NT10, and NFL for several indicators, whereas CMA, NT2, and NT4 had lower scores for many of the selected indicators. Overall, the integrated score showed that the strongest relative performance occurred mainly in the longer‐duration no‐till systems, especially NT10, with clearer separation in the 0–5 cm layer than in the 5–10 cm layer.

## 4 Discussion

### 4.1 Carbon–Nitrogen Dynamics and Organic Matter Stabilization

The contrasting land‐use systems showed distinct carbon–nitrogen responses, but the magnitude of change differed among soil pools and depths. Total organic carbon showed moderate variation in the 0–5 cm layer, whereas clearer differences occurred in the 5–10 cm layer, where natural forest and longer‐duration no‐till systems had higher values than conventionally managed arable land. This pattern suggests that longer‐duration no‐till‐based conservation management influenced near‐surface carbon distribution, likely through continuous organic inputs, surface mulch, perennial root turnover and reduced mechanical disruption. Similar responses have been reported in tropical and subtropical systems where conservation management improves soil organic matter accumulation by reducing oxidation and increasing residue retention (Lal 2004; Blanco‐Canqui and Lal 2008; Tyagi et al. 2019; Usharani et al. 2019).

Nitrogen responded more consistently than carbon along the no‐till chronosequence. Total nitrogen and nitrogen stock increased from NT2 to NT10, indicating greater nitrogen retention under longer‐duration conservation management. This is mechanistically plausible in humid tropical soils, where high rainfall, rapid organic matter turnover, and low inherent fertility can accelerate nitrogen losses through leaching, erosion, and mineralization (Belmonte et al. 2018). Poultry manure, mulch, and perennial crop cover likely supplied organic nitrogen, while reduced disturbance favored microbial immobilization and physical protection of organic N within aggregates. This agrees with previous tropical studies showing that organic inputs and reduced disturbance can improve nitrogen retention when conservation practices are sustained over time (Franzluebbers 2002; Chivenge et al. 2022; Mesele et al. 2026).

Carbon and nitrogen stocks showed similar broad trends, with higher surface‐layer stocks under NT7 and NT10. This suggests that sustained conservation management can rebuild nutrient capital in shallow, highly weathered tropical soils. The relatively high subsoil carbon stock under natural forest highlights the role of long‐term vegetation cover, litter cycling, root inputs, and minimal disturbance in maintaining organic matter below the immediate surface layer (Don et al. 2011; Mesele and Huising 2024). The C:N ratio further reflected differences in organic matter quality and nitrogen enrichment. A higher C:N ratio under conventionally managed arable land indicates lower nitrogen status relative to carbon, while lower C:N ratios under several no‐till and forest soils suggest more nitrogen‐enriched organic matter pools. However, the C:N ratio is an indicator of relative carbon–nitrogen balance, not as a direct measure of mineralization.

Organic matter fractions revealed a staged recovery pathway. High particulate organic carbon under conventionally managed arable land may reflect residue incorporation by tillage, but this pool is dynamic and more exposed to decomposition. In contrast, the increase in particulate organic carbon and nitrogen with no‐till duration, especially in the surface layer, indicates gradual rebuilding of active organic matter pools under sustained residue return and reduced disturbance (Cambardella and Elliott 1992; Wander and Yang 2000). Non‐particulate organic carbon and nitrogen were less linear across the chronosequence, but their higher values under longer‐duration no‐till and natural forest systems suggest greater contribution of stabilized organic matter pools. In low‐activity tropical soils, such stabilization depends strongly on continuous inputs, microbial transformation, organo‐mineral association, and physical protection within aggregates (Denef et al. 2007; Cotrufo et al. 2013; Yang et al. 2025).

### 4.2 Soil Structure, Water Regulation and Nutrient Retention

Aggregate stability was one of the clearest indicators of improved soil physical condition under longer‐duration no‐till‐based management. The highest values under NT10 suggest that repeated organic inputs, surface cover, root activity, and reduced mechanical disturbance promoted aggregate formation and persistence. This mechanism is important in Ferric Luvisols and related tropical soils, where coarse surface texture, weak structure, and intense rainfall can increase crusting, runoff, and erosion. Improved aggregation can protect organic matter, improve pore continuity and reduce the loss of nutrient‐rich particles (Bronick and Lal 2005; Blanco‐Canqui and Lal 2008). Bulk density did not follow the same pattern as aggregate stability. This is expected because no‐till does not always reduce bulk density, especially during early transition stages when mechanical loosening has stopped, but biological porosity is still developing. The higher bulk density under NT2 may reflect residual compaction or early‐stage structural adjustment, whereas the lower bulk density under natural forest is consistent with continuous litter input, root activity, and biological pore formation (Blanco‐Canqui et al. 2011; Soane et al. 2012). Thus, soil physical recovery is better captured by combining bulk density with indicators of aggregation and water retention.

Water‐regulation indicators showed greater functional improvement under NT7 and NT10. Higher available water‐holding capacity and field capacity indicate greater retention of water in plant‐available pore spaces, probably due to organic matter accumulation, improved aggregation, mulch cover, and reduced surface evaporation. This is particularly relevant in tropical agroecosystems where dry spells can occur despite high annual rainfall. Conservation management can improve soil water buffering by increasing organic matter and stabilizing soil structure (Franzluebbers 2002; Govaerts et al. 2007; Abdallah et al. 2021; Li et al. 2025). Lower leaching potential under NT7 and NT10 links the structural and hydrological responses to nutrient retention. In coarse‐textured, low‐CEC tropical soils, nutrient leaching is a major pathway of fertility loss. Organic matter improves exchange sites, aggregation, and water retention, thereby reducing rapid nutrient displacement. This pattern agrees with Mesele et al. (2026), who reported lower leaching potential and stronger nutrient retention in longer‐established conservation systems on similar Ferric Luvisols.

Chemical fertility indicators reinforced this functional improvement. Higher soil pH under NT10 may reflect the buffering effect of organic amendments and the return of base cations from manure and plant residues. Higher available phosphorus under NT10 likely reflects organic nutrient inputs, surface accumulation, and reduced erosion losses, whereas higher phosphorus under conventional management reflects mineral fertilizer use. The increase in cation exchange capacity under NT10 is particularly significant because organic matter substantially contributes to nutrient retention in highly weathered soils dominated by kaolinite and quartz. Improved CEC, therefore, indicates a greater capacity to retain cations and buffer nutrient losses (Karlen et al. 2003; Lal 2004; Lehmann et al. 2020).

### 4.3 Integrated Soil Health Response and Land‐Use Contrasts

The integrated soil health score showed the strongest overall performance under NT10, followed by NT7. This pattern reflects the combined improvements in nitrogen accumulation, carbon and nitrogen stocks, aggregate stability, water retention, and cation exchange capacity (Dai et al. 2026). The contrast between NT2 and NT10 is particularly important because it shows that short‐term conversion did not immediately improve all soil functions. Instead, broad soil health improvement became more evident after several years of continuous organic input, soil cover, and reduced disturbance. This agrees with tropical conservation agriculture studies showing that soil recovery is gradual and depends on sustained residue return, organic nutrient inputs, biological activity, and erosion protection (Thomas et al. 2007; Aziz et al. 2013; Chivenge et al. 2022; Song et al. 2026).

Natural forest served as a useful reference, but it did not consistently have the highest values for all indicators. This is expected because forest soils and managed conservation systems represent different functional states. Forest soils rely on long‐term litter cycling and minimal disturbance, while NT7 and NT10 received manure, mulch, irrigation, and perennial crop cover. The longer‐duration no‐till systems are not approaching forest conditions; rather, they represent managed conservation systems that can exceed forest values on some fertility and water‐regulation indicators due to external organic inputs and active management. The PCA and correlation analyses supported the multidimensional nature of the soil health response. Associations among aggregate stability, water‐retention indicators, pH, and cation exchange capacity suggest that soil structure, water regulation, and nutrient retention are linked. This is mechanistically coherent: organic inputs support aggregation; aggregation improves pore organization; improved pore organization enhances water retention; and organic matter increases exchange capacity. These linked pathways explain why the strongest soil health response occurred where carbon–nitrogen pools, aggregation, water retention, and nutrient‐retention capacity improved together.

### 4.4 Scope and Implications

Three points define the scope of inference. First, the no‐till systems were separate fields established in different years, so duration effects represent chronosequence associations rather than repeated measurements from the same plots over time. Second, no‐till was combined with poultry manure, mulch, residue cover, irrigation, and perennial crop cover, while the conventional system involved tillage, annual crops, and mineral fertilizer use. The observed differences, therefore, reflect integrated no‐till‐based conservation management, not the isolated effect of tillage exclusion. Third, sampling was limited to the upper 10 cm because of iron pan and stone concretions, so the findings mainly describe surface and near‐surface soil responses.

Despite these constraints, the study shows that sustained no‐till‐based conservation management can improve several linked dimensions of soil function in tropical agroecosystems. The strongest responses occurred in nitrogen accumulation, carbon and nitrogen stocks, aggregate stability, water retention, pH, phosphorus availability, cation exchange capacity, and the integrated soil health score. These findings support long‐term, integrated conservation management as a practical pathway for improving soil function, reducing nutrient‐loss risk, and strengthening resilience in highly weathered tropical agricultural soils.

## 5 Conclusions

Our findings support the hypothesis that longer‐duration no‐till‐based systems are associated with improved soil carbon–nitrogen status and soil functional indicators compared with conventionally managed arable land and shorter‐duration no‐till systems. The strongest responses were observed under NT7 and NT10, particularly for total nitrogen, nitrogen stock, carbon stock, aggregate stability, available water‐holding capacity, field capacity, pH, available phosphorus, cation exchange capacity, and the integrated soil health score. These improvements indicate that sustained organic inputs, surface cover, reduced disturbance and perennial crop management can progressively strengthen nutrient retention, water regulation and structural stability in highly weathered tropical soils. The response was not uniform across all indicators, indicating that soil recovery proceeds through multiple pathways, with nitrogen enrichment and improvements in soil physical–chemical functioning emerging more clearly than those for some carbon fractions. Natural forest provided an important reference condition, but the longer‐duration no‐till systems did not simply replicate forest soil conditions. Instead, they represented a distinct managed conservation system in which manure input, mulching, residue cover, irrigation, and reduced disturbance jointly improved selected soil functions. Therefore, the observed effects are system‐level responses to no‐till‐based conservation management rather than the isolated effect of no‐till alone. Overall, the study demonstrates that sustained conservation‐oriented management can rebuild key components of soil health in tropical agroecosystems, especially within the surface and near‐surface soil layers. These results highlight the importance of long‐term implementation when using no‐till‐based systems as a strategy for restoring soil function, improving nutrient retention, and strengthening the resilience of degraded or intensively managed tropical agricultural lands.

## Funding

The authors have nothing to report.

## Conflicts of Interest

The authors declare no conflicts of interest.

## Supporting information


**Table S1:** Chronosequence trend slopes for soil carbon–nitrogen pools, stocks and soil health indicators across no‐till systems.
**Table S2:** Detailed particulate organic carbon and nitrogen fractions under contrasting land‐use systems and soil depths.
**Figure S1:** The study area highlighting the sampling points at the various land use types.
CMA, Conventionally managed arable land; NFL, Natural forest land; NT, no‐till system.
**Figure S2:** Particle size distribution across contrasting land‐use systems and soil depths. Panels show normalized (a) sand, (b) silt, and (c) clay fractions expressed as percentages of total measured particle‐size fractions within each sample. Bars represent means and error bars indicate standard errors. Particle size distribution is presented as a supplementary site‐characterization result and was not interpreted as a management‐induced soil health response. CMA, conventionally managed arable land; NT2, 2‐year no‐till system; NT4, 4‐year no‐till system; NT7, 7‐year no‐till system; NT10, 0‐year no‐till system; NFL, natural forest land.
**Figure S2:** Supplementary physical indicators across contrasting land‐use systems. Panels show (a) rooting depth and (b) leaching potential. Bars represent means and error bars indicate standard errors. Different letters indicate significant differences among land‐use systems based on block‐adjusted Tukey comparisons at *p* < 0.05. CMA, conventionally managed arable land; NT2, 2‐year no‐till system; NT4, 4‐year no‐till system; NT7, 7‐year no‐till system; NT10, 10‐year no‐till system; NFL, natural forest land.

## Data Availability

The dataset supporting the findings of this study has been deposited in Zenodo and is publicly available at https://doi.org/10.5281/zenodo.20796006. The dataset should be cited as follows: Mesele, S., Babatunde, A., & Uponi, J. (2026). *Soil C‐N pools and soil health indicators across different land use systems in Guinea Savanna ecology of Nigeria* [Data set]. Zenodo. https://doi.org/10.5281/zenodo.20796006.
