# Supplementary material for: Soil Carbon–Nitrogen Pools and Soil Health Across Contrasting Land Uses and a No‐Till Conservation Chronosequence in a Tropical Guinea Savanna Agroecosystem
Source: Plant Environ Interact. 2026 Jul 17;7(4):e70190. doi: 10.1002/pei3.70190 (PMC13377525; doi:10.1002/pei3.70190)
Supplement: Supplementary file 1 — Table S1: Chronosequence trend slopes for soil carbon–nitrogen pools, stocks and soil health indicators across no‐till systems. Table S2: Detailed particulate organic carbon and nitrogen fractions under contrasting land‐use systems and soil depths. Figure S1: The study area highlighting the sampling points at the various land use types. CMA, Conventionally managed arable land; NFL, Natural forest land; NT, no‐till system. Figure S2: Particle size distribution across contrasting land‐use systems and soil depths. Panels show normalized (a) sand, (b) silt, and (c) clay fractions expressed as percentages of total measured particle‐size fractions within each sample. Bars represent means and error bars indicate standard errors. Particle size distribution is presented as a supplementary site‐characterization result and was not interpreted as a management‐induced soil health response. CMA, conventionally managed arable land; NT2, 2‐year no‐till system; NT4, 4‐year no‐till system; NT7, 7‐year no‐till system; NT10, 0‐year no‐till system; NFL, natural forest land. Figure S2: Supplementary physical indicators across contrasting land‐use systems. Panels show (a) rooting depth and (b) leaching potential. Bars represent means and error bars indicate standard errors. Different letters indicate significant differences among land‐use systems based on block‐adjusted Tukey comparisons at p < 0.05. CMA, conventionally managed arable land; NT2, 2‐year no‐till system; NT4, 4‐year no‐till system; NT7, 7‐year no‐till system; NT10, 10‐year no‐till system; NFL, natural forest land. [file PEI3-7-e70190-s001.docx]

**Supplementary material**

**Table S1. Chronosequence trend slopes for soil carbon–nitrogen pools, stocks and soil health indicators across no-till systems**

| Indicator | 0–5 cm slope (95% CI) | p-value | 5–10 cm slope (95% CI) | p-value |
| --- | --- | --- | --- | --- |
| TOC (g kg⁻¹ yr⁻¹) | 0.126 (−0.114, 0.365) | 0.272 | 0.300 (0.061, 0.539) | 0.019 |
| TN (g kg⁻¹ yr⁻¹) | 0.243 (0.087, 0.399) | 0.006 | 0.381 (0.225, 0.537) | <0.001 |
| POCt (10^-1^ g C yr⁻¹) | 0.879 (0.272, 1.486) | 0.009 | 0.088 (−0.520, 0.695) | 0.756 |
| PONt (10^-1^ g N yr⁻¹) | 3.518 (2.575, 4.462) | <0.001 | 0.073 (−0.870, 1.017) | 0.867 |
| nPOC (g C yr⁻¹) | 0.283 (−0.475, 1.041) | 0.428 | 0.332 (−0.426, 1.090) | 0.356 |
| nPON (10^-1^ g N yr⁻¹) | 0.117 (−0.398, 0.632) | 0.627 | 0.140 (−0.375, 0.655) | 0.561 |
| C stock (Mg ha⁻¹ yr⁻¹) | 0.205 (0.096, 0.314) | 0.002 | 0.069 (−0.040, 0.178) | 0.193 |
| N stock (Mg ha⁻¹ yr⁻¹) | 0.091 (0.025, 0.156) | 0.012 | 0.169 (0.103, 0.235) | <0.001 |
| C:N (yr⁻¹) | 0.917 (−0.349, 2.182) | 0.139 | 0.077 (−1.188, 1.342) | 0.896 |
| Aggregate stability (% yr⁻¹) | 0.677 (0.278, 1.075) | 0.003 | 0.829 (0.430, 1.227) | <0.001 |
| Bulk density (g cm⁻³ yr⁻¹) | −0.003 (−0.039, 0.033) | 0.863 | 0.007 (−0.029, 0.043) | 0.675 |
| AWHC (mm m⁻¹ yr⁻¹) | 0.446 (−0.007, 0.898) | 0.053 | 0.637 (0.185, 1.090) | 0.010 |
| Field capacity (mm m⁻¹ yr⁻¹) | 0.017 (−0.004, 0.039) | 0.108 | 0.036 (0.014, 0.058) | 0.004 |
| Permanent wilting point (mm m⁻¹ yr⁻¹) | −0.005 (−0.025, 0.015) | 0.603 | −0.001 (−0.021, 0.020) | 0.958 |
| Ks (cm h⁻¹ yr⁻¹) | 0.144 (−0.626, 0.914) | 0.688 | −0.519 (−1.290, 0.251) | 0.166 |
| Soil pH (unit yr⁻¹) | 0.147 (0.100, 0.193) | <0.001 | 0.127 (0.080, 0.173) | <0.001 |
| EC (S m⁻¹ yr⁻¹) | 11.406 (9.434, 13.379) | <0.001 | 4.278 (2.306, 6.251) | <0.001 |
| Available P (mg kg⁻¹ yr⁻¹) | 2.437 (1.032, 3.842) | 0.003 | 1.295 (−0.109, 2.700) | 0.067 |
| CEC (cmolc kg⁻¹ yr⁻¹) | 0.457 (0.141, 0.773) | 0.009 | 0.463 (0.147, 0.778) | 0.008 |
| Rooting depth (cm yr⁻¹) | 1.007 (0.555, 1.459) | <0.001 | 0.238 (−0.214, 0.690) | 0.271 |
| Leaching potential (% yr⁻¹) | −0.981 (−1.582, −0.379) | 0.004 | −1.293 (−1.895, −0.692) | <0.001 |

Slopes represent the estimated change in each indicator per additional year of no-till duration, calculated using NT2, NT4, NT7 and NT10 only. Values in parentheses are 95% confidence intervals. Positive slopes indicate an increase with no-till duration, while negative slopes indicate a decrease. TOC = total organic carbon; TN = total nitrogen; POCt = total particulate organic carbon; PONt = total particulate nitrogen; nPOC = non-particulate organic carbon; nPON = non-particulate nitrogen; AWHC = available water-holding capacity; Ks = saturated hydraulic conductivity; EC = electrical conductivity; CEC = cation exchange capacity.

**Table S2. Detailed particulate organic carbon and nitrogen fractions under contrasting land-use systems and soil depths**

| Soil layer | Land use | POCc  ………………..…10^-1^ x grams………………….… | PONc | POCf | PONf |
| --- | --- | --- | --- | --- | --- |
| 0–5 cm | CMA | 7.0ᵃ | 4.0ᵇ | 8.0ᵃ | 8.9ᵇ |
| 0–5 cm | NT2 | 0.1ᵇ | 0.3ᶠ | 3.7ᵇ | 0.9ᵉ |
| 0–5 cm | NT4 | 0.1ᵇ | 2.1ᶜ | 0.6ᶜ | 4.8ᵈ |
| 0–5 cm | NT7 | 0.1ᵇ | 1.7ᵈ | 2.2ᵇᶜ | 7.9ᶜ |
| 0–5 cm | NT10 | 1.2ᵇ | 1.4ᵉ | 9.3ᵃ | 30.1ᵃ |
| 0–5 cm | NFL | 6.2ᵃ | 4.7ᵃ | 8.3ᵃ | 0.9ᵉ |
| 0–5 cm | **p-value** | <0.001 | <0.001 | <0.001 | <0.001 |
| 5–10 cm | CMA | 0.1ᵃ | 0.6ᵇ | 2.4ᵇ | 1.8ᶜ |
| 5–10 cm | NT2 | 0.1ᵃ | 0.1ᵈ | 0.4ᶜ | 1.8ᶜ |
| 5–10 cm | NT4 | 0.3ᵃ | 0.1ᵈ | 2.5ᵇ | 1.0ᵉ |
| 5–10 cm | NT7 | 0.1ᵃ | BLDᵉ | 2.1ᵇᶜ | 2.5ᵇ |
| 5–10 cm | NT10 | 0.1ᵃ | 0.5ᶜ | 1.6ᵇᶜ | 1.4ᵈ |
| 5–10 cm | NFL | 2.4ᵃ | 0.7ᵃ | 4.9ᵃ | 4.5ᵃ |
| 5–10 cm | **p-value** | 0.519 | <0.001 | 0.002 | <0.001 |

Values are means. Within each soil layer and variable, means followed by the same superscript letter are not significantly different based on block-adjusted Tukey comparisons at p < 0.05. The p-value rows indicate the land-use effect within each soil depth from block-adjusted ANOVA. CMA = conventionally managed arable land; NT2 = 2-year no-till system; NT4 = 4-year no-till system; NT7 = 7-year no-till system; NT10 = 10-year no-till system; NFL = natural forest land; POCc = coarse particulate organic carbon; PONc = coarse particulate nitrogen; POCf = fine particulate organic carbon; PONf = fine particulate nitrogen; BLD = below detection limit.


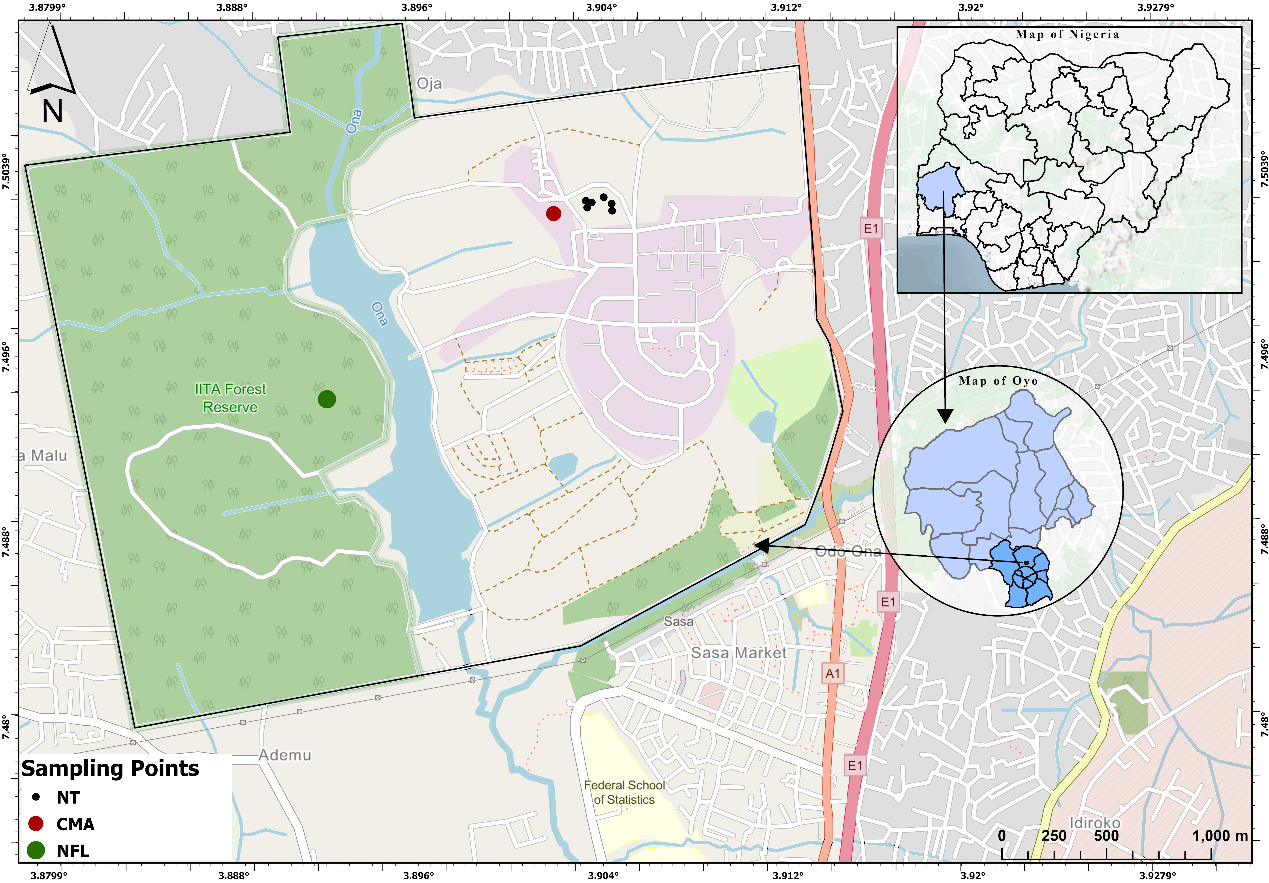


**Figure S1: The study area highlighting the sampling points at the various land use types.**

CMA =Conventionally managed arable land; NFL =Natural forest land; NT =no-till system.

**
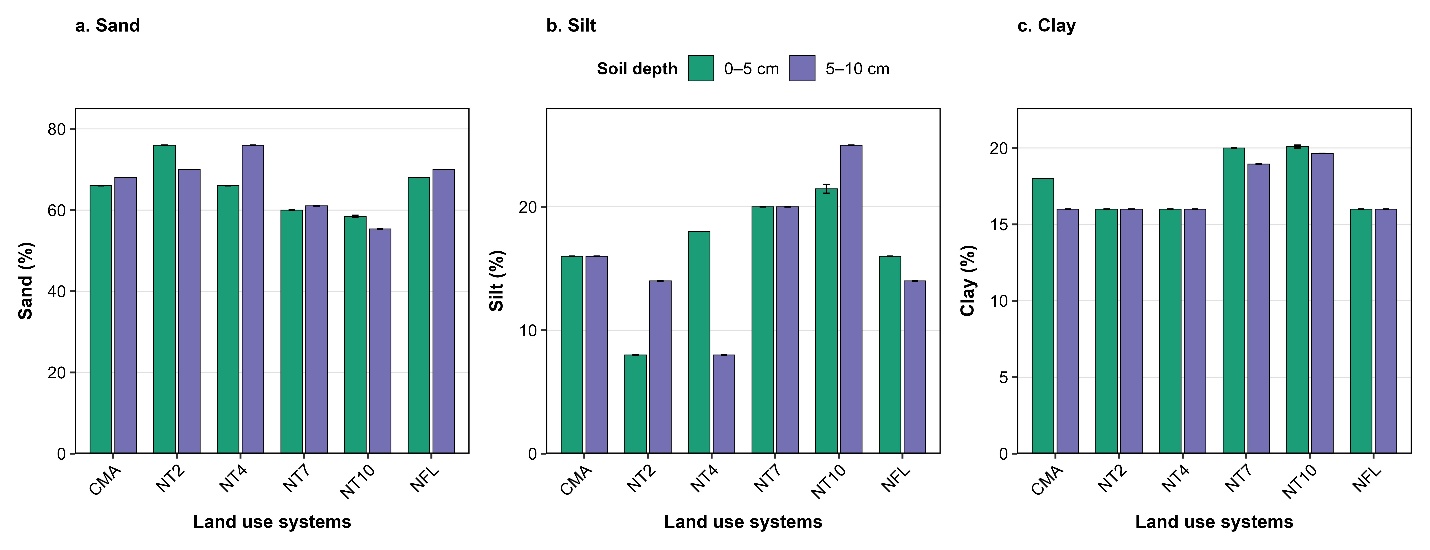
**

**Figure S2. Particle size distribution across contrasting land-use systems and soil depths.** Panels show normalized (a) sand, (b) silt, and (c) clay fractions expressed as percentages of total measured particle-size fractions within each sample. Bars represent means and error bars indicate standard errors. Particle size distribution is presented as a supplementary site-characterization result and was not interpreted as a management-induced soil health response. CMA = conventionally managed arable land; NT2 = 2-year no-till system; NT4 = 4-year no-till system; NT7 = 7-year no-till system; NT10 = 10-year no-till system; NFL = natural forest land.


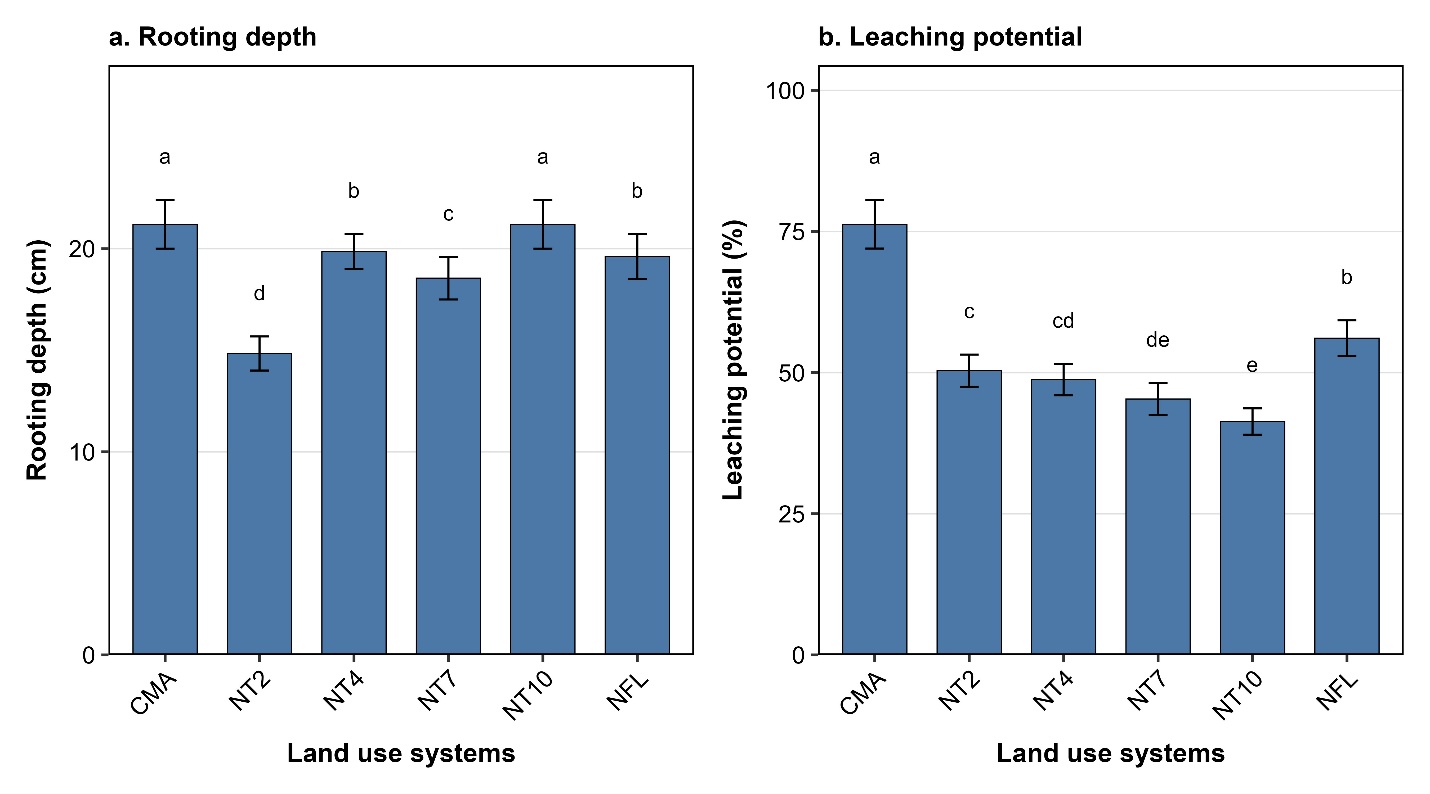


**Figure S2. Supplementary physical indicators across contrasting land-use systems.** Panels show (a) rooting depth and (b) leaching potential. Bars represent means and error bars indicate standard errors. Different letters indicate significant differences among land-use systems based on block-adjusted Tukey comparisons at p < 0.05. CMA = conventionally managed arable land; NT2 = 2-year no-till system; NT4 = 4-year no-till system; NT7 = 7-year no-till system; NT10 = 10-year no-till system; NFL = natural forest land.
